# MDSC-decreasing chemotherapy increases the efficacy of cytokine-induced killer cell immunotherapy in metastatic renal cell carcinoma and pancreatic cancer

**DOI:** 10.18632/oncotarget.6734

**Published:** 2015-12-23

**Authors:** Zibing Wang, Yuqing Liu, Yong Zhang, Yiman Shang, Quanli Gao

**Affiliations:** ^1^ Department of Immunotherapy, Henan Cancer Hospital and Affiliated Cancer Hospital of Zhengzhou University, Zhengzhou 450008, China; ^2^ Department of Oncology, Third Affiliated Hospital of Xinxiang Medical College, Xinxiang 453003, China

**Keywords:** myeloid-derived suppressor cells, cytokine-induced killer cells, immunotherapy, solid tumors, overall survival

## Abstract

Adoptive immunotherapy using cytokine-induced killer (CIK) cells is a promising cancer treatment, but its efficacy is restricted by various factors, including the accumulation of myeloid-derived suppressor cells (MDSCs). In this study, we determine whether chemotherapeutic drugs that reduce MDSC levels enhance the efficacy of CIK cell therapy in the treatment of solid tumors. Fifty-three patients were included in this study; 17 were diagnosed with metastatic renal cell carcinoma (MRCC), 10 with advanced pancreatic cancer (PC), and 26 with metastatic melanoma (MM). These patients were divided into two groups: CIK cell therapy alone and CIK cell therapy combined with chemotherapy. Combining CIK cell therapy and chemotherapy increased 1-year survival rates and median survival times in MRCC and PC patients, but not in MM patients. The disease control rate did not differ between treatment groups for MRCC or MM patients, but was higher in PC patients receiving combined treatment than CIK cell treatment alone. These data suggest that addition of MDSC-decreasing chemotherapy to CIK cell therapy improves survival in MRCC and PC patients.

## INTRODUCTION

Although surgical intervention after an early diagnosis is highly effective, the prognoses for metastatic renal cell carcinoma (MRCC), metastatic melanoma (MM), and pancreatic cancer (PC) remain poor. The median survival time of MRCC patients is 10 months [1], and the 5-year survival rate is less than 10% [2]. For MM patients, the median survival time is 8 to 9 months, and the 3-year survival rate is less than 15% [3]. PC patients have the lowest survival by stage of any solid tumor [4]. The development of new therapies, including VEGF axis inhibitors for MRCC and a BRAF inhibitor for MM, has helped improve survival rates in advanced patients. However, these drugs rarely induce a complete and enduring response. Checkpoint inhibitors, such as monoclonal antibodies against cytotoxic T-lymphocyte-associated antigen 4 and programmed death 1, represent a breakthrough in the treatment of solid tumors, including MRCC and MM [5, 6]. However, their high cost limits their clinical use, especially in developing countries.

At present, chemotherapy is still a common therapeutic approach for MRCC, MM and PC, although its efficacy is limited [7, 8]. For example, only 5% of advanced PC patients respond to treatment with gemcitabine and their median overall survival is only 5.7 months [9]. A clinical trial of combined gemcitabine/erlotinib treatment showed only a slight improvement, which increased median survival to 6.2 from the 5.9 months seen with gemcitabine alone [10]. In comparison to gemcitabine, FOLFIRINOX (oxaliplatin, irinotecan, fluorouracil, and leucovorin) or nab-paclitaxel plus gemcitabine therapy increased median overall survival by 4.3 and 1.8 months, respectively [11, 12]. However, these combined treatments are associated with a higher incidence of serious side effects, and patients must therefore undergo rigorous testing prior to chemotherapy and closer monitoring during treatment.

Since the discovery that interleukin-2 (IL-2) administration can benefit MM patients, the importance of tumor immunotherapy as a cancer treatment has grown [13, 14]. Rosenberg and colleagues found that adoptive cell transfer utilizing autologous tumor infiltrating lymphocytes helped 20 of 93 pretreated MM patients (22%) achieve a lasting complete response [15]. Implantation of cytokine-induced killer (CIK) cells has been a particularly promising adoptive immunotherapy, and their anti-tumor characteristics have proven clinically effective in treating many solid tumors [16–18]. In MRCC patients, adoptive transfer of CIK cells prolonged survival compared to combined IL-2 and interferon (IFN) α treatment [19]. However, additional research is needed to improve the efficacy of CIK cell treatments.

We previously found that CD11b^+^Gr-1^+^ myeloid-derived suppressor cells (MDSCs) inhibit anti-tumor immune responses and reduce the efficacy of immunotherapy in an animal model [20, 21]. In a clinical setting, low CD11b^+^CD33^+^HLA-DR^−^ MDSC levels in peripheral blood were associated with improved prognosis in MRCC patients receiving CIK cell therapy [22]. Reducing MDSC levels delays tumor growth and prolongs survival [23, 24]. Recent studies demonstrate that conventional chemotherapeutic agents, such as gemcitabine and 5-fluorouracil, kill tumor-associated MDSCs [25, 26]. Tyrosine kinase inhibitors such as sunitinib also decrease MDSC levels in patients with renal cell carcinoma [27]. In addition, promoting differentiation of MDSCs into mature, non-suppressive cells using all-*trans* retinoic acid (ATRA) reduces MDSC levels in MRCC patients [28].

In this study, we retrospectively analyzed clinical data from 17 MRCC, 10 advanced PC and 26 MM patients to determine whether administration of chemotherapeutic agents enhanced the efficacy of CIK cell therapy. We also examined MDSC levels to determine whether they were decreased after chemotherapy drugs were used.

## RESULTS

### Patients

MRCC, PC, and MM patient characteristics are listed in Tables 1, 2 and 3, respectively. The groups were similar with respect to age, sex, and extent of disease. All patients had metastatic disease and Karnofsky performance status scores greater than 80.

### MDSC levels before and after chemotherapy drug administration

**Figure 1:**
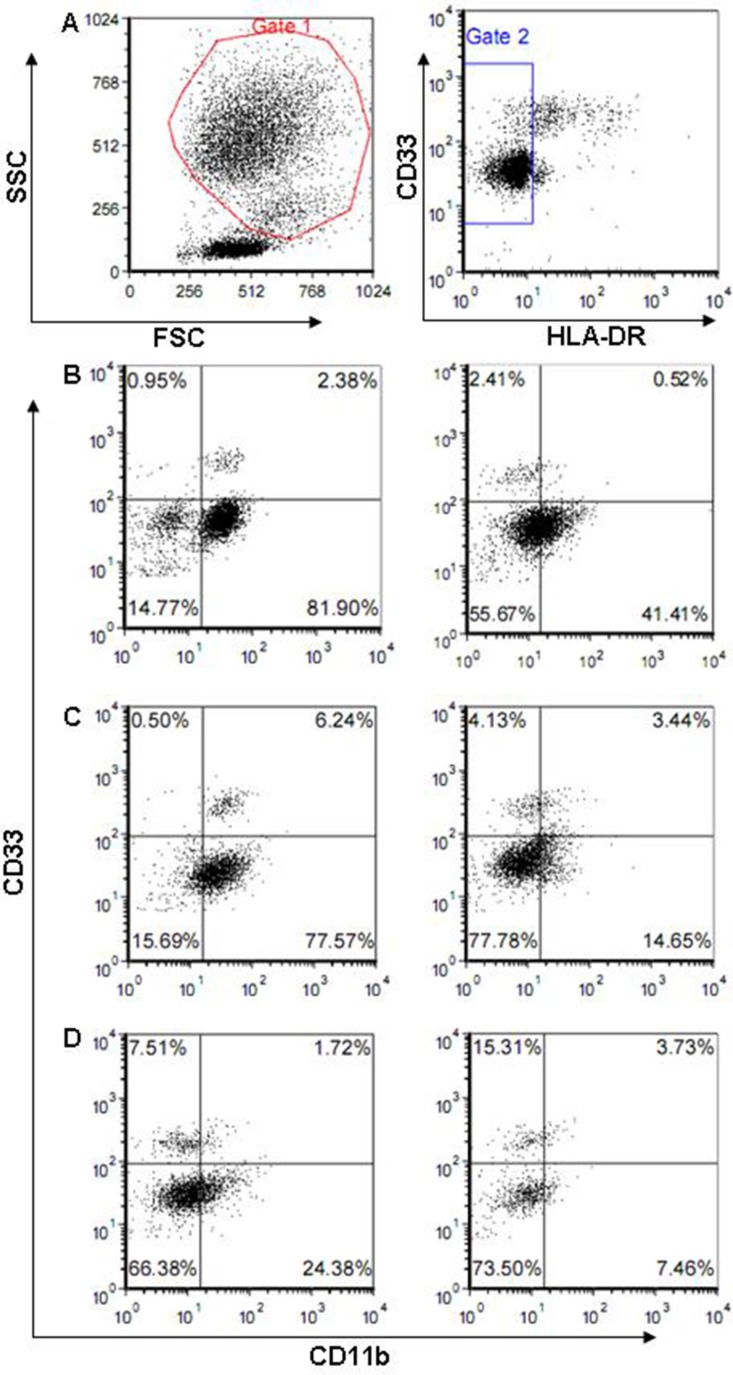
Flow cytometric analysis of peripheral blood mononuclear cells by FACScan Data shown are for one representative MRCC (**B**), PC (**C**), and MM (**D**) patient before (left) and after (right) administration of 5-fluorouracil, gemcitabine, and dacarbazine, respectively. HLA-DR^−^ cells detected in region 2 were divided into four fractions based on the expression of CD11b and CD33. CD11b^+^CD33^+^ cells were analyzed. Panel A shows the gate used to analyze MDSCs.

Both 5-fluorouracil and gemcitabine prevent MDSC accumulation in tumor-bearing hosts [25, 26], while dacarbazine does not [29]. We confirmed these results by examining MDSC levels in the peripheral blood of cancer patients. Representative MDSC levels before and after chemotherapy are shown in Figure 1 for representative MRCC, MM, and PC patients.

### Survival and response

**Figure 2:**
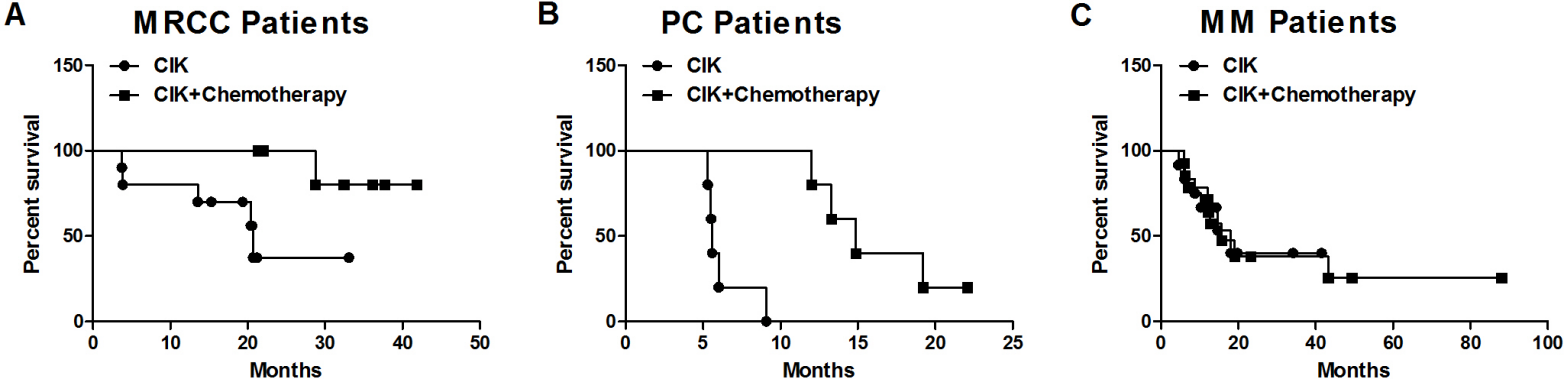
Survival curve for MRCC (**A**), PC (**B**), and MM (**C**) patients receiving CIK cell therapy alone and CIK cell therapy combined with chemotherapy.

**Figure 3:**
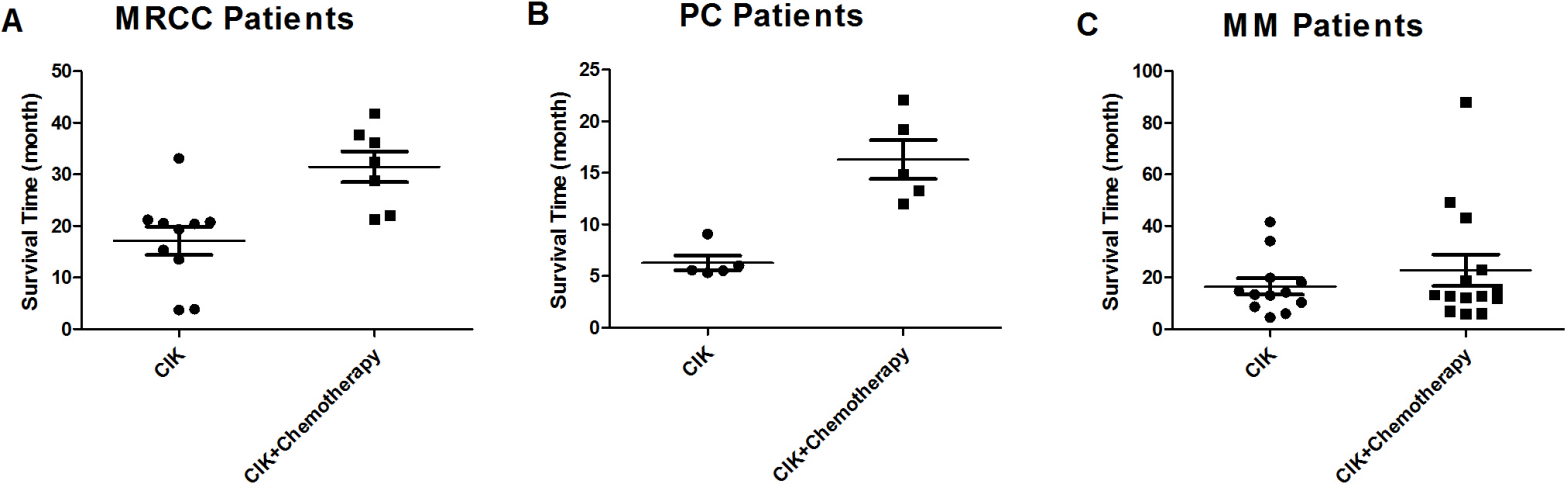
Median survival times for MRCC (**A**), PC (**B**), and MM (**C**) patients receiving CIK cell therapy alone and CIK cell therapy combined with chemotherapy.

There were differences in survival between treatment groups in MRCC and PC patients. Kaplan-Meier curves for survival by treatment type are shown in Figure 2. One-year survival rates increased from 80% in MRCC patients receiving only CIK cell therapy to 100% in patients receiving both CIK cell treatment and chemotherapy (*p* = 0.035). At the completion of patient monitoring, 37% of CIK-treated MRCC patients were alive compared to 80% of patients receiving CIK cell treatment and chemotherapy (*p* = 0.035; Figure 2A). CIK-treated MRCC patients survived a median of 19.9 months (range: 3.7 to 33.1 months) after the first CIK cell infusion, while the median survival in patients receiving both CIK cell treatment and chemotherapy was 32.4 months (range: 21.3 to 41.9 months) (*p* = 0.0035; Figure 3A). Similarly, CIK-treated PC patients had lower 1-year survival rates (*p* = 0.002; Figure 2B) and median survival times (*p* = 0.001; Figure 3B) (0% and 5.6 months, range: 5.3 to 9.1) than PC patients treated with both CIK cells and chemotherapy (80% and 14.9 months, range: 12.0 to 22.1). However, there were no treatment-dependent differences in 1-year survival rate or median survival time (66.7% versus 71.4% (*p* = 0.92) and 13.9 versus 13.1 months (*p* = 0.39); Figures 2C and 3C) in MM patients.

**Figure 4:**
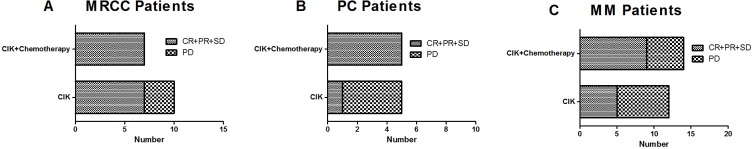
Treatment response rates of MRCC (**A**), PC (**B**), and MM (**C**) patients receiving CIK cell therapy alone and CIK cell therapy combined with chemotherapy.

Objective response rates did not differ depending on treatment in MRCC patients or MM patients. The disease control rate (DCR) was 70% in CIK-treated MRCC patients and 100% in CIK- and chemotherapy-treated patients (*p* = 0.23, Table 1 and Figure 4A). The DCRs following the same treatments in MM patients were 42% and 64%, respectively (*p* = 0.23, Table 3 and Figure 4C). In PC patients, however, DCRs were higher in those receiving CIK cells and chemotherapy than in those receiving only CIK cell treatment (100% and 20%, respectively, *p* = 0.048, Table 2 and Figure 4B).

**Table 1:** Characteristics of the MRCC patients

Patient No	Age/Sex	Historical type	Metastases	Therapy	Outcome
1	54/M	Chromophobe	Lung, bone	Sunitinib + CIK	PR, alive 32.4 months
2	78/M	Clear cell	Lung, LN	Chemotherapy + CIK	SD, alive 21.3 months
3	54/F	Clear cell	Liver	Chemotherapy + CIK	PR, alive 22.0 months
4	63/F	Clear cell	Lung, bone	Chemotherapy + CIK	SD, alive 37.7 months
5	71/M	Clear cell	Lung	Chemotherapy + CIK	SD, alive 36.1 months
6	46/M	Clear cell	Lung	Chemotherapy + CIK	SD, alive 41.9 months
7	64/F	Clear cell	Lung	ATRA + CIK	SD, died on month 28.8
8	65/M	Clear cell	Lung	CIK	PR, alive 19.4 months
9	48/M	Clear cell	Brain, Lung, LN	CIK	PD, died on month 13.5
10	70/M	Clear cell	Lung, LN	CIK	SD, alive 15.3 months
11	78/M	Clear cell	Lung	CIK	SD, died on month 20.4
12	72/M	Clear cell	Lung	CIK	SD, alive 21.2 months
13	55/F	Clear cell	Lung, liver	CIK	SD, alive 33.1 months
14	50/M	Clear cell	Liver, pancrea, LN	CIK	PD, died on month 3.9
15	21/F	Clear cell	Lung, liver, bone, LN	CIK	PD, died on month 3.7
16	54/M	Papillary	Lung	CIK	SD, alive 20.6 months
17	49/M	Clear cell	Lung	CIK	SD, died on month 20.7

Abbreviations: LN, lymph nodes

**Table 2:** Characteristics of the PC patients

Patient No	Age/Sex	Diagnosed date	Metastases	Therapy	Outcome
1	70/M	2010.09.01	Lung	Chemotherapy + CIK	SD, died on month 12.0
2	58/M	2011.09.07	Abdominal LN	Chemotherapy + CIK	SD, died on month 13.3
3	49/F	2011.09.07	Liver	Chemotherapy + CIK	SD, died on month 14.9
4	72/M	2011.05.21	Liver	Chemotherapy + CIK	PR, died on month 19.2
5	52/M	2011.06.1	Abdominal LN	Chemotherapy + CIK	SD, alive on 22.1 months
6	75/F	2010.10.16	Mesenterium	CIK	SD, died on month 9.1
7	66/M	2012.04.20	Liver	CIK	PD, died on month 5.3
8	65/M	2012.06.2	Bone, AC, PC	CIK	PD, died on month 5.5
9	43/F	2012.07.1	Lung, Liver	CIK	PD, died on month 5.6
10	61/F	2012.07.13	Liver	CIK	PD, died on month 6.0

Abbreviations: LN, lymph nodes; AC, abdominal cavity; PC, pelvic cavity

**Table 3:** Characteristics of the MM patients

Patient No	Age/Sex	Diagnosed date	Metastases	Therapy	Outcome
1	55/M	2010.07.05	Lung, pleura, LN	Chemotherapy + CIK	PR, died on month 7.0
2	45/M	2007.08.22	Lung, liver, bone	Chemotherapy + CIK	PD, died on month 43.3
3	37/F	2012.04.13	Liver	Chemotherapy + CIK	SD, alive on 13.4 months
4	48/F	2010.04.10	Liver, LN	Chemotherapy + CIK	SD, died on month 19.0
5	53/M	2011.09.21	Bone, LN	Chemotherapy + CIK	SD, died on month 6.2
6	47/F	2006.01.15	Bone, Skin	Chemotherapy + CIK	CR, died on month 88.0
7	50/F	2011.05.01	Lung, Spleen	Chemotherapy + CIK	PD, died on month 12.0
8	77/M	2012.05.02	Lung	Chemotherapy + CIK	SD, alive on 12.8 months
9	55/F	2009.04.10	Lung, Liver	Chemotherapy + CIK	PD, alive on 49.3 months
10	48/F	2010.03.12	Lung, Bone, LN	Chemotherapy + CIK	SD, died on month 12.2
11	47/M	2011.09.15	Lung, Liver, Bone, PC	Chemotherapy + CIK	PD, died on month 6.0
12	64/F	2012.04.11	Lung, Liver, Skin	Chemotherapy + CIK	PR, died on month 12.8
13	26/M	2011.06.09	Lung, Liver, Bone, Adrenaline	Chemotherapy + CIK	PD, died on month 15.6
14	38/M	2011.06.23	LN	Chemotherapy + CIK	PR, alive on 23.1 months
15	51/F	2011.09.30	Brain, LN, Spleen	CIK	SD, alive on 19.8 months
16	53/M	2009.07.23	Lung, LN, PC	CIK	PD, Died on month 41.5
17	76/M	2011.12.30	LN	CIK	PD, Died on month 4.5
18	55/F	2010.07.26	Skin	CIK	SD, alive on 34.1 months
19	76/F	2012.04.12	LN	CIK	SD, alive on 13.4 months
20	29/M	2012.04.25	LN	CIK	SD, alive on 13.0 months
21	44/F	2011.05.24	Vagina, LN	CIK	PD, died on month 18.0
22	55/M	2011.10.26	Liver, Stomach	CIK	PD, died on month 6.1
23	66/F	2012.03.16	Lung, LN	CIK	SD, alive on 14.3 months
24	48/F	2011.04.14	Nasal cavity	CIK	PD, died on month 8.7
25	76/F	2011.07.01	Skin, LN	CIK	PD, died on month 10.3
26	67/F	2012.02.09	Lung, Skin	CIK	PD, died on month 14.5

Abbreviations: LN, lymph nodes; PC, pelvic cavity

### Adverse effects

More severe treatment toxicity resulted from combined CIK cell treatment and chemotherapy than from CIK cell treatment alone. Specifically, combined treatment resulted in lower blood counts and increases in nonhematologic events, including nausea and vomiting, diarrhea, skin reactions, nerve changes, fatigue, and oral mucositis. The most common adverse effect of CIK cell therapy alone was fever, which occurred in approximately 15% patients. These patients recovered from this side effect either without treatment or after the oral administration of non-steroidal anti-inflammatory drugs such as indomethacin [22].

## DISCUSSION

Our results suggest that combining chemotherapy with immunotherapy might improve survival in MRCC and PC patients. Recently, it was reported that combining gemcitabine and/or S-1 chemotherapy with dendritic cell vaccine immunotherapy prolongs median survival to 12 months [30], while the median survival was 8.8–10.1 months when these chemotherapy drugs were not combined with the immunotherapy (31). Here, we found that PC patients receiving CIK cell therapy combined with gemcitabine had a median survival time of 14.9 months as compared to 5.6 months in those receiving CIK cell treatment alone. In MRCC patients, combined CIK cell and chemotherapy treatment resulted in a 1-year survival rate of 100% as compared to 80% in those receiving only CIK cell therapy. MRCC patients receiving CIK cell treatment alone survived a median of 19.9 months, while those receiving CIK cell treatment and chemotherapy survived 32.4 months, suggesting that combining these therapies improves survival.

Furthermore, we found that decreased MDSC levels were associated with the superior efficacy of this combined therapy. CIK cells are mainly CD3^+^CD56^+^ cells, which express CD4 and CD8 and have natural killer (NK) cell activity [32–34]. The tumoricidal activity of CIK cells partly depends on their ability to produce IFNγ [35]. NKG2D-NKG2D ligand interactions, which are necessary for NK cell activation and cytolytic activity, also contribute to the antitumor activity of CIK cells [36]. It is thus possible that CIK cells exert antitumor effects by reducing the activity of immune inhibitory factors that target T and NK cells. MDSC levels increase dramatically during tumor progression, and MDSCs inhibit both T cells and NK cells in animal models and in cancer patients [37–39]. Therefore, depletion of MDSCs may represent a new approach to cancer immunotherapy. The commonly used chemotherapy drugs 5-fluorouracil, gemcitabine, sunitinib and ATRA all effectively decrease MDSC levels [25–28]. As expected, we show here that administration of these drugs to MRCC and PC patients receiving CIK cell immunotherapy prolonged survival time. Similar clinical observations in patients with other solid tumors have been reported recently. Combining chemotherapy (5-fluorouracil and oxaliplatin) with CIK cell treatment in patients with advanced gastric cancer improves quality of life and increases 2-year survival rate compared to treatment with chemotherapy alone [40]. Increased treatment efficacy has also been observed in advanced non-small cell lung cancer patients when docetaxel, which is also known to eliminate MDSCs, is combined with CIK cell therapy [41, 42].

Combining chemotherapy and immunotherapy may not improve outcomes for all types of cancer, however. Here, CIK-treated MM patients had similar median survival times with or without chemotherapy. This result may be due in part to the use of dacarbazine, a cytotoxic drug widely used for melanoma treatment, which does not affect T regulatory cells (Tregs), MDSC levels, or dendritic cell maturation [29]. It is possible that combining CIK cell therapy with a different MDSC-decreasing drug instead might improve survival in MM patients as well. A recent report found that chemotherapy-induced lymphodepletion is essential before cell transfer in MM patients because it eliminates immune inhibitory cells (e.g. Tregs or MDSCs) from the tumor microenvironment [43]. We are currently carrying out a clinical trial to evaluate the efficacy of combined docetaxel and CIK cell treatment in MM patients.

In addition to their direct cytotoxic effects on tumor cells, chemotherapy drugs likely improve survival by enhancing CIK cell immunotherapy. A recent study reported that gemcitabine and 5-fluorouracil do more than simply induce MDSC cell death. These two drugs appear to also activate the pro-apoptotic protein Bax (Bcl2-associated X protein) in MDSCs, which induces two separate molecular cascades. The first activates the production and release of pro-tumorigenic cytokines, eventually reducing anticancer immunity and promoting tumor growth. The second cascade induces MDSC apoptosis, thus enhancing the antitumor efficacy of these drugs [44]. Another recent report evaluated the effects of dacarbazine on antitumor immune responses. Although no change in MDSC levels was observed, dacarbazine triggered the upregulation of NKG2D ligands on tumor cells, leading to NK cell activation and IFNγ secretion. NK cell-derived IFNγ upregulates major histocompatibility complex class I molecules on tumor cells, rendering them sensitive to cytotoxic CD8^+^ T cells [29]. These examples of the immunogenic effects of chemotherapy drugs further suggest that combining them with immunotherapies can improve cancer prognoses.

In contrast to murine models, a distinct and widely accepted marker combination for human MDSCs is still not available due to the heterogeneous nature of these immature cells, even within a single tumor type. For example, HLA-DR^−^Lin1^low/−^CD33^+^CD11b^+^ cells are designated MDSCs in patients with PC [45]. However, in another report, CD33^+^HLADR^−^CD11b^+^CD15^+^ cells and CD33^+^HLADR^−/low^CD14^+^ cells were proposed as MDSCs, and both were elevated in PC [46]. Interestingly, recent research suggests that levels of two out of five antigens examined (HLA-DR and CD33, but not CD11b, CD14, or CD15) provide an accurate estimate of overall MDSC levels in PC [47]. Similarly, in MM patients, CD14^+^HLA-DR^neg/low^ cells, CD14^+^IL4Ralpha^+^ cells, Lin^−^HLA-DR^−^CD33^+^ cells, CD15^+^IL4Ralpha^+^ cells, and CD14^−^CD66b^+^Arginase1^+^ cells were categorized as MDSCs [48–51]. We previously reported that, in MRCC patients, CD11b^+^CD33^+^HLA-DR^−^ cells were MDSCs and their levels were correlated with patient outcome [22]. Thus, several populations of MDSCs have been identified, and it is likely that multiple populations are present in the peripheral blood mononuclear cells of patients with a single tumor type. In the present study, we defined MDSCs as CD11b^+^CD33^+^HLA^−^ cells in MRCC, PC and MM. However, detailed investigation of specific MDSC subsets and their immunosuppressive activities in patients will help to improve cancer treatments.

Future studies should address two key limitations of the present experiments. First, the retrospective, non-randomized nature of this study, which included patients from only one hospital, may limit the generalizability of the results. Survival differences between patients receiving CIK cell treatment alone and in combination with chemotherapy might be affected by differences between groups during patient selection and differences in standard and supportive-care treatment. Second, the inclusion of a third patient group receiving only chemotherapy would have strengthened the results. However, the results of a previous retrospective analysis of 82 advanced PC patients suggest that combining CIK cell therapy with chemotherapy increased overall survival as compared to chemotherapy treatment alone. Together, these studies provide strong evidence that treatment efficacy increases when immunotherapy and chemotherapy are combined.

## MATERIALS AND METHODS

### Patients

Between March 19, 2010, and July 13, 2012, we enrolled 53 histologically-confirmed cancer patients from Henan Cancer Hospital & Affiliated Cancer Hospital of Zhengzhou University. 17 had MRCC with lung/liver metastases, 10 had advanced PC, and 26 had metastatic MM. All patients were separated into two cohorts. Patients in the first cohort received CIK cell therapy alone (CIK-treated); those in the second cohort received CIK cell therapy and chemotherapy. Eligible patients were at least 18 years of age and had a life expectancy of at least 12 weeks. All patients gave written informed consent, which was approved by the institutional review board of Zhengzhou University. This study was conducted in accordance with the provisions of the Declaration of Helsinki and Good Clinical Practice guidelines.

### Treatment

Of the 17 MRCC patients, 10 patients were given CIK cell therapy alone and 7 were treated with CIK cell therapy combined with chemotherapy. The chemical agents used for the combined treatment included 2 cases of 5-fluorouracil (400 mg/m^2^, ivgtt, d1–5), 2 cases of gemcitabine (1000 mg/m^2^, ivgtt, d1, 8), 1 case of 5-fluorouracil plus gemcitabine, 1 case of sunitinib (50 mg, po, d1–28), and 1 case of ATRA (45 mg/m^2^, po, d1–28). Patients were given 1 cycle of 5-fluorouracil and/or gemcitabine every 3 weeks. After they completed all chemotherapy cycles (usually 2–6 cycles), these patients began to receive CIK cell therapy. For cases treated with oral sunitinib and ATRA, CIK cells were transfused concomitantly with chemotherapy drugs.

Of the 10 PC patients, 5 patients were given CIK cell therapy alone and 5 were given CIK cell therapy combined with gemcitabine-based chemotherapy (1000 mg/m^2^, ivgtt, d1, 8). Of the patients receiving combined treatment, 3 cases received CIK cell therapy after completing 2–6 cycles of chemotherapy (sequential combination treatment). The other 2 patients were given cycles of chemotherapy followed 2 days later by CIK cell transfusion (concomitant combination treatment); this treatment cycle was repeated 6 times.

Of the 26 MM patients, 12 were given CIK cell therapy alone and 14 were given CIK cell therapy combined with dacarbazine-based chemotherapy (300 mg/m^2^, ivgtt, d1–5). Of the patients receiving combined treatment, 5 received 2–6 cycles of sequential combination treatment, and the rest received 2–6 cycles of concomitant combination treatment.

CIK cell suspensions were prepared as described previously [22]. Briefly, peripheral blood mononuclear cells were separated and cultured under sterile conditions in 1640 medium containing anti-CD3 monoclonal antibody, IFNγ, IL-2, and RetroNectin (RN, Takara, Japan). After culturing the cells for 10 to 14 days, a target dose of about 5×10^9^ CIK cells with over 95% viability was obtained and tested for biological contaminants. Cells were then prepared in sodium chloride solution containing 2% albumin before transfusion. After transfusion, patients were given IL-2 (two million IU per day) for 3 days to promote CIK cell activity. Patients in this study received at least 2 cycles of CIK cell transfusions.

For analysis of MDSCs, peripheral blood cells from MRCC, PC, and MM patients were collected aseptically by venipuncture, treated with BD FACS Lysing solution to lyse erythrocytes, stained with anti-CD33, anti-CD11b, and anti-HLA-DR monoclonal antibodies, and analyzed by flow cytometry. Peripheral blood was collected from each patient on 2 occasions. The first collection was done before chemotherapy and the second was done 15 days after chemotherapy was administered.

### Evaluation of short-term efficacy and toxicity

Tumor response was separated into four categories according to the Response Evaluation Criteria in Solid Tumors (RECIST): complete response (CR), partial response (PR), stable disease (SD), and progressive disease (PD). CR, PR, and SD were included in the disease control rate (DCR). Tumors were assessed using computed tomographic (CT) scanning at baseline and 2 months after the initiation of treatment. Safety was assessed by examining records of adverse events. Hematologic and serum chemical measurements were performed before and after each cycle of treatment. Adverse events were graded using the Common Terminology Criteria for Adverse Events of the National Cancer Institute, version 3.0.

### Statistical analysis

The statistical probability of survival was calculated according to the method of Kaplan and Meier. Differences between outcomes were compared using Log-rank (Mantel-Cox) Test. MDSC levels and survival times were compared between groups using unpaired *t*-tests. Fisher's exact *t*-test was used to compare short-term efficacy between groups.
